# Effect of Spermidine on Misfolding and Interactions of Alpha-Synuclein

**DOI:** 10.1371/journal.pone.0038099

**Published:** 2012-05-25

**Authors:** Alexey V. Krasnoslobodtsev, Jie Peng, Josephat M. Asiago, Jagadish Hindupur, Jean-Christophe Rochet, Yuri L. Lyubchenko

**Affiliations:** 1 Department of Pharmaceutical Sciences, University of Nebraska Medical Center, Omaha, Nebraska, United States of America; 2 School of Medicine, Shanghai Jiao Tong University, Shanghai, People's Republic of China; 3 Department of Medicinal Chemistry and Molecular Pharmacology, Purdue University, West Lafayette, Indiana, United States; University of South Florida College of Medicine, United States Of America

## Abstract

Alpha-synuclein (α-Syn) is a 140 aa presynaptic protein which belongs to a group of natively unfolded proteins that are unstructured in aqueous solutions. The aggregation rate of α-Syn is accelerated in the presence of physiological levels of cellular polyamines. Here we applied single molecule AFM force spectroscopy to characterize the effect of spermidine on the very first stages of α-Syn aggregation – misfolding and assembly into dimers. Two α-Syn variants, the wild-type (WT) protein and A30P, were studied. The two protein molecules were covalently immobilized at the C-terminus, one at the AFM tip and the other on the substrate, and intermolecular interactions between the two molecules were measured by multiple approach-retraction cycles. At conditions close to physiological ones at which α-Syn misfolding is a rare event, the addition of spermidine leads to a dramatic increase in the propensity of the WT and mutant proteins to misfold. Importantly, misfolding is characterized by a set of conformations, and A30P changes the misfolding pattern as well as the strength of the intermolecular interactions. Together with the fact that spermidine facilitates late stages of α-Syn aggregation, our data demonstrate that spermidine promotes the very early stages of protein aggregation including α-Syn misfolding and dimerization. This finding suggests that increased levels of spermidine and potentially other polyamines can initiate the disease-related process of α-Syn.

## Introduction

Alpha-synuclein (α-Syn) is an abundant presynaptic protein, the aggregation of which is a hallmark of several neurodegenerative diseases, collectively called synucleopathies including Parkinson's disease [1]. Structurally α-Syn can be divided into three distinct domains: (i) the N-terminal amphipathic domain, which comprises the first 60 amino acids; (ii) the hydrophobic NAC (non-amyloid component) region, which spans residues 61–95; and (iii) the C-terminal domain spanning residues 96–140, which is negatively charged at neutral pH. Alpha-synuclein is commonly accepted to be a natively unfolded protein (NUP), which means that it lacks secondary/tertiary structure in aqueous solutions [2], [3]. Although unstructured, α-Syn undergoes a structural transformation when it binds to lipids of membranes and vesicles, adopting α-helical structure primarily within the N-terminal domain and the NAC region, while C-terminus remains free and unstructured [4]. Structural transitions are also involved in the conversion of α-Syn to amyloid-like fibrils with a high content of ß-sheet secondary structure both *in vivo* and *in vitro*.

Many factors have been found to promote aggregation of α-Syn. For example, single point mutants of the protein (A30P [5], E46K [6] and A53T [7]) are found in early-onset familial Parkinson's disease and they also accelerate aggregation of α-Syn *in vitro* [8]. Posttranslational modifications of alpha-synuclein such as phosphorylation [9] and oxidation [10] have also been found to alter aggregation propensity and toxicity of the protein. Truncation of the C-terminus [11] and alternative splicing [12] depriving alpha-synuclein of the C-terminal part also accelerate aggregation. Environmental factors promote the aggregation of α-Syn. The conditions include low solution pH values [13], elevated temperature [13] and the presence of multivalent metal ions [14], [15]. Importantly, it has been recently reported that the metabolic pathway of polyamines contributes to PD pathogenesis *in vivo* [16]. Reduced activity of the catabolic enzyme SAT1 increases cellular levels of higher order polyamines (spermine/spermidine) which in turn enhances toxicity of α-Syn. These results suggest that polyamines in a cellular context are directly involved in PD pathology at physiological concentrations [16]. Consequently, understanding the structural basis of monomeric α-Syn and its self-association in the presence of polyamines is of particular importance.

In this work we used a novel experimental approach that directly probes interactions between aggregation-prone (misfolded) states of proteins [17], [18], [19]. This approach utilizes Atomic Force Microscopy (AFM) force spectroscopy capable of direct measurements of interactions between individual alpha-synuclein molecules. In this approach, two α-Syn molecules are covalently immobilized via the C-terminus, one at the AFM tip and the other on the substrate, and intermolecular interactions between the two protein molecules are measured by multiple approach-retraction cycles. This technique applied to a number of proteins including α-Syn revealed a critical role of dimerization in the protein misfolding and further aggregation process [19]. Here, we used this probing technique to directly characterize the role of spermidine in the misfolding of α-Syn. We demonstrate that spermidine promotes the α-Syn misfolding process and characterize the misfolding states of the protein. Moreover, the single protein mutation A30P characteristic of familial PD changes the pattern of the protein misfolding. These findings along with prior studies indicate a very strong effect of spermidine on all steps of α-Syn aggregation and suggest that metabolism of spermidine can be a factor critically involved in PD development.

## Results

### Experimental design and approach

The covalent attachment of protein molecules to the AFM probe and the mica substrate is the key factor for reliable and reproducible single molecule AFM force spectroscopy [19]. We took advantage of the fact that the α-Syn sequence lacks any cysteine residues and created alpha-synuclein variants with cysteine instead of alanine at the C-terminal position 140. The single cysteine residue allowed us to covalently attach proteins by their C terminus to the AFM tip and mica surface using surface chemistry as described in [18], [19], [20], [21], [22]. Briefly, AFM tips and mica surface were treated with aqueous solution of maleimide silatrane (MAS) which provides modification of surfaces with maleimide functional groups [20]. Next, α-Syn A140C variants are attached to the surface via the reaction of maleimide and the – SH group of cysteine. The protein immobilization was done in a freshly prepared working protein solution in PBS pretreated with DTT to minimize dimerization of α-Syn via disulfide bond formation (as described in methods section). Note that the concentration of the protein in the working solution was much less (by ∼ 3 orders of magnitude) than the concentration of protein used in aggregation experiments *in vitro* [18]. The use of diluted protein prevents aggregation of α-Syn during the surface immobilization process and ensures the attachment of monomeric species to the surfaces. The low concentration of working solution also resulted in a sparse distribution of the protein on the surface minimizing simultaneous multiple interactions [22].

We chose the end of the C-terminus as the point for the covalent attachment because the aggregation related structural changes in α-Syn are limited to the N-terminal and NAC regions of the protein [4]. These regions are also responsible for the transition to an alpha-helical structure and association with lipids [4]. The NAC region was found in fibrils and accommodates ß-sheet secondary structure [23]. On the other hand, the C-terminal region of α-Syn is highly acidic and proline-rich and has been suggested to be in a fully random conformation in both the free and lipid bound forms of the protein [4]. Based on this fact we have used only a short linker within MAS (5 units of ethylene glycol) anticipating that the C-terminal region of α-Syn would act as a flexible tether for force spectroscopy experiments.

### Single molecule force measurements

**Figure 1:**
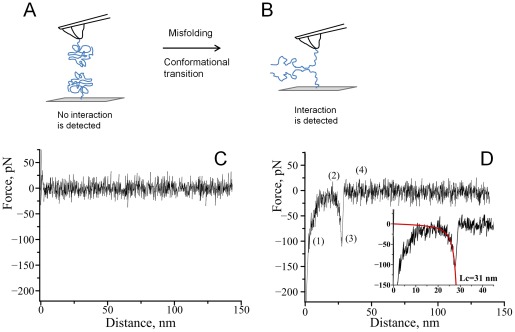
Schematic representation of the experimental approach. A) The unstructured form of alpha-synuclein immobilized on the tip and a substrate reveals no interaction. B) Misfolding of alpha-synuclein results in the formation of aggregation-prone conformation with elevated propensity of intermolecular interactions. C) A representative force-distance curve measured in the absence of spermidine. D) A representative force –distance curve with a rupture event in the presence of spermidine: (1) an adhesion peak due to short-range non-specific interactions between the tip and the surface, (2) gradual increase in force characteristic of polymer stretching, (3) complete rupture at 110 pN and (4) region where tip comes free from the surface. The inset shows worm-like chain fitting yielding contour length of 31 nm.

To understand the role of polyamines in the first step of the aggregation process – dimer formation between α-Syn – we have measured interactions between individual α-Syn molecules in multiple approach-retraction cycles in PBS in the presence and absence of spermidine. Misfolding of alpha-synuclein results in a conformational transition of α-Syn which favors interactions between the two molecules, as shown in Fig. 1A, B. Interactions between covalently attached molecules are probed in multiple approach-retraction cycle on various different spots of the surface. Such cycling allows a molecule on the AFM probe to interact with its partner upon approach with the formation of dimer. If no stable dimeric contact is formed the retraction of the tip does not detect any rupture event (Fig. 1C). However, a stable dimeric contact produces a specific rupture signature (Fig. 1D) in the force-distance curve. The beginning of such a force curve usually contains an adhesion peak (1) due to short-range non-specific interactions between the tip and the surface. The adhesion peak is followed by a complete rupture (3) where the tip comes free from the surface (4). The complete rupture is preceded by a part characteristic of the stretching of polymer linkers (2). We have fitted this part of the curve with a worm like chain (WLC) model describing the behavior of α-Syn under an external force. Such a fit gives parameters for the expected fully stretched C-terminal part of α-Syn that does not participate in interprotein interactions.

### Effect of spermidine on misfolding of α-Syn

**Figure 2:**
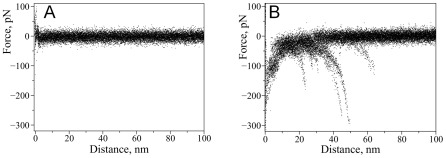
Effect of spermidine on the appearance of force-distance curves with wild-type alpha-synuclein. Superposition of representative force-distance curves measured upon probing of interactions between wild type alpha-synuclein molecules: A) in the absence of spermidine (22 curves), B) with addition of 5 mM spermidine (26 curves).

Probing of α-Syn interactions at neutral pH (PBS buffer) did not reveal rupture events suggesting that no stable dimeric contact is formed between alpha-synuclein molecules at these conditions. This is supported by Fig. 2A which shows a superposition of a number of representative force curves measured in PBS. No specific rupture events are evident on these curves. This observation is consistent with our previous force spectroscopy studies [22] suggesting that misfolding of α-Syn and interactions between monomers is a very rare event as no rupture events were found at these conditions.

Next, we repeated probing experiments in the presence of 5 mM spermidine. For that we have replaced the buffer in the cell with the one containing spermidine and continued measuring force-distance curves with the same tip and substrate. The effect of spermidine on the appearance of the force-distance curves was dramatic. Force distance curves with well defined rupture events similar to the one shown in Fig. 1D appeared. Superposition of representative force-distance curves measured in the presence of spermidine is shown in Fig. 2B. For WT α-Syn the yield of measurable specific rupture events was 1.8%.

**Figure 3:**
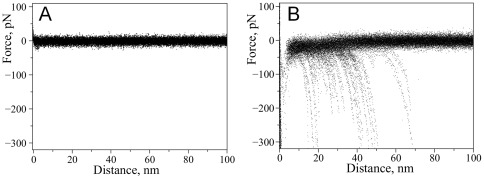
Effect of spermidine on the appearance of force-distance curves with A30P alpha-synuclein. Superposition of representative force-distance curves measured upon probing of interactions between A30P alpha-synuclein molecules: A) in the absence of spermidine (40 curves), B) with addition of 5 mM spermidine (58 curves).

We performed a similar study with a single point mutant A30P of α-Syn for which an early onset of PD was observed [24]. The results are shown in Fig. 3. Similar to WT α-Syn, we did not observe any specific interactions for A30P in PBS without spermidine while in the presence of 5 mM spermidine specific rupture events were observed. Figure 3 shows superpositions of representative force-distance curves in the absence (Fig. 3A) and presence of spermidine (Fig. 3B). The yield of rupture events was 1.9% and it is comparable to the yield measured for WT indicating that the effect of spermidine for both WT and A30P mutant is rather similar. Previously, using this AFM force spectroscopy approach we detected a strong effect of divalent cations and low pH on α-Syn misfolding [18], [21], [22]. Qualitatively, the effect of spermidine is similar to the one observed for metal cations since both induce misfolding of α-Syn [22].

### Misfolded states of α-Syn in the presence of spermidine

**Figure 4:**
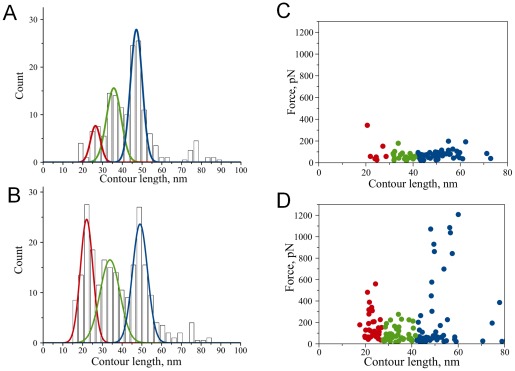
Statistical histograms of measured contour length values. A) Wild type, B) A30P mutant; the histograms were fit with Gaussian functions resulting in the following values of distribution maxima: 26 nm, 36 nm, 47 nm for WT and 22 nm, 34 nm, 49 nm for A30P mutant. Rupture force versus contour length plots C) Wild type, D) A30P mutant.

As illustrated by Fig. 1D, AFM force spectroscopy in our single molecule setup with a well-defined tethering of the proteins provides two parameters characterizing specific rupture events – rupture force and rupture length. The rupture force defines the strength of the dimer, whereas the rupture length determines the position of the protein segment involved in the interprotein interaction. Comparison of Figs. 2B and 3B shows that the rupture length distributions for the WT α-Syn and A30P mutant are quite different. The force curves for the WT α-Syn are grouped with the primary rupture events around 50 nm whereas rupture events for the mutant are rather uniformly distributed between ∼30 nm and ∼70 nm. We developed the rupture length analysis approach allowing us to localize segments of the protein involved in dimer formation [18], [21], [25]. Here we used this approach to characterize structural differences of misfolded WT and A30P α-Syn. For each force-distance curve with a specific rupture event, we determined the contour length by fitting a characteristic rupture signature with a worm-like chain model. A statistical histogram of measured contour length values is shown in Fig. 4A for WT α-Syn. The Gaussian fit of the statistical histogram resulted in maxima of distributions at 26, 36 and 47 nm defined as peaks 1, 2 and 3 respectively. The occurrence of several peaks on the distribution suggests that there is more than one conformation of WT α-Syn within the dimer. The results of a similar analysis for the A30P mutant are shown in Fig. 4B. The distribution also has three clear maxima located at 22, 34 and 49 nm as determined from the Gaussian fit. Rupture peaks cluster at around the same distances as for WT. The contour length distribution, however, is wider for the mutant than for WT suggesting that despite similarities these molecules are quite different in their behavior. Some of the rupture peaks appear at shorter distances with the maximum of *peak 1* being smaller than for WT. Additionally, the ratio between the peaks is quite different with a greater contribution from the shortest peak (*peak 1*) for the A30P mutant.

Rupture forces also point to differences in the nature of monomer-monomer interactions for these two molecules. Fig. 4C and D show a plot of rupture forces versus contour length for WT and A30P respectively. Whereas the distribution of rupture forces for WT α-Syn is rather narrow around 60 pN, the distribution of rupture forces for A30P mutant is broader with the appearance of rupture events as large as ∼1.2 nN. The stronger rupture forces can explain a higher propensity of A30P to aggregate compared to WT α-Syn.

**Figure 5:**
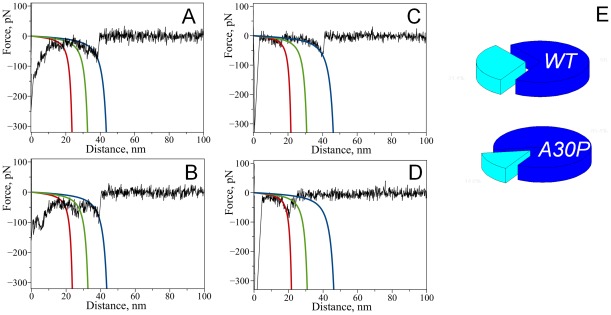
Complex force-distance curves with multiple rupture peaks detected with WT alpha-synuclein (A, B) and A30P mutant (C, D). Red, green and blue lines on the graphs are calculated WLC curves using maxima of Gaussians determined from contour length distributions corresponding to 26, 36 and 47 nm (WT) and 22, 34 and 49 nm (A30P). E) Pie charts showing relative contribution of multiple rupture events to total rupture events: 31% (WT) and 15% (A30P).

Although the majority of the events were observed with a single rupture that corresponds to the breaking of a dimeric complex between individual alpha-synuclein molecules [22], [26], we have observed complex force-distance curves with more than one rupture peak. Figure 5 shows a set of representative force-distance curves with multiple ruptures observed for wild type (Fig. 5A, B) and A30P (Fig. 5C, D) α-Syn. Each of them has multiple rupture peaks approximated with WLC curves with different colors. Each of these events is located approximately at the same positions, but the rupture force values are different. For example, the middle transition (green curves) has the largest rupture value in panel B whereas a similar transition in panel A is much weaker. The positions of the multiple ruptures were within the 20–70 nm region coinciding with the region observed for single events. Force-distance curves with multiple rupture events suggest that interactions between individual alpha-synuclein molecules have a more complex nature than one would describe by a simple one-site dimeric contact. Force-distance curves with multiple events have a minor contribution to the overall yield of rupture events compared to the single rupture events. The A30P mutant showed similar behavior, with the majority of the force-distance curves having single rupture peaks. Although curves with multiple rupture events were observed for both WT and A30P mutant, the yield of multiple rupture events was two-fold greater in the case of the WT protein (31% versus 15%; Fig. 5E). Additionally, complexity of the force distance curves with multiple ruptures differed for these two variants as indicated by the representative curves on Fig. 5. Majority of the curves with multiple rupture events for A30P had two ruptures while WT usually had three ruptures per curve.

## Discussion

### Structure of misfolded α-Syn within the dimers

This single molecule AFM force spectroscopy study clearly shows a strong effect of spermidine on α-Syn misfolding. Moreover, with this approach we are able to characterize misfolded states of the protein. This information can be retrieved from the contour length measurements using the approach we developed earlier [18].

**Figure 6:**
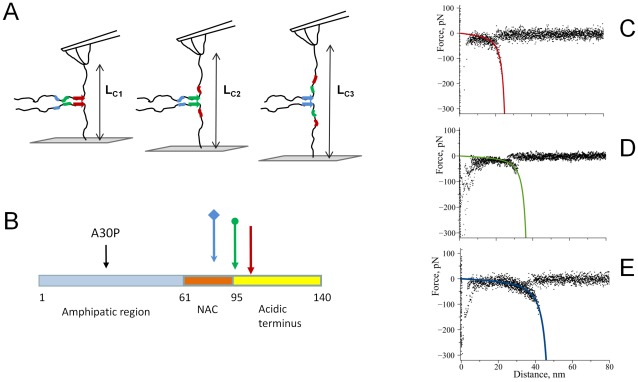
Interaction model of alpha-synuclein molecules. The model describes three major peaks in the contour length histograms as identical sites in each monomer interacting with each other with the formation of a dimer. A) Position of the interacting site further from the C-terminus (point of attachment) results in longer contour length value. B) Positions at the beginning of the detected interaction sites. Colored arrows correspond to three detected interaction sites schematically shown in A), and the black arrow shows the position of the A30P mutation in alpha-synuclein; C), D) and E) superposition of representative force-distance curves for the detected rupture events corresponding to L_C1_, L_C2_ and L_C3_, red, green and blue lines on the graphs are WLC curves calculated with L_C_ values from Fig. 4.

The rupture events appear at a specific distance on the force-distance curve defining the length of the flexible linkers. For our experimental design these flexible linkers consist of the C-terminal portion of the α-Syn molecule not involved in misfolding and interprotein interaction. The experimentally measured distributions of contour lengths for both proteins (Figs 4A, B) have clear maxima suggesting the existence of preferred interactions. Each maximum on the distribution corresponds to a certain length of stretchable, free C-terminal regions of interacting α-Syn molecules. Figure 6 schematically shows the model of interactions between α-Syn molecules. The positions of three main peaks, *p1*, *p2* and *p3*, correspond to *L_C1_*, *L_C2_* and *L_C3_* values on the contour length distribution. In order to estimate the relative position of interacting sites we have assumed a symmetric model [18], [21], [25]. This symmetric model assumes the same identity of interacting sites within both α-Syn molecules. The length of the C-terminal part is then half the value of L_C_ after subtracting the length of the short MAS linker [18], [20]. The experimentally determined contour length value (L_C_) consists of the length of the linkers and stretchable segment of the protein. The latter was determined by subtracting known values of the linkers' length from the L_C_ value. For example, peak *p3* with *L_C3_*=47 nm would correspond to lengths of two extended C-terminal parts of the protein 47 nm–3 nm (length of 2 MAS linkers) =44 nm or 22 nm per one C-terminus. Given the interaminoacid distance (0.34 nm) this value leads to the C-terminus length of 65 aa. The position of the interacting region is then 140–65=75 aa.

Rupture lengths of the force peaks clustered into three main reproducible interactions occurring at 26, 36 and 47 nm for WT alpha-synuclein. Given that an 11 aa segment of the NAC region comprising residues (73–83) is the most dominant segment determining α-Syn's ability to form fibrils [23], [27] we expected a contour length of 48 nm when the C-terminus is completely stretched. Our measured distribution indeed features such a peak, and the majority of events are observed at this length at least for WT. However, two additional interactions are also observed that take place at shorter distances, 36 and 26 nm corresponding to a rupture at 96 aa and 110 aa, respectively. While the 96 aa interaction still falls within the C-terminal part of the NAC region (which spans residues 61 to 95), the position of the shortest interaction (110 aa) extends far into the C-terminal region. Such shortening of rupture lengths has also been observed in the presence of multivalent cations [22]. Importantly, the pathologic mutant A30P showed an increased ratio of these short interactions. The following are possible explanations for this effect: (1) the interacting regions which are capable of forming dimers extend beyond the NAC region into the C-terminal part, (2) the C-terminal region is not fully released from long range-interactions [28], (3) polyamines bridge two alpha-synuclein molecules. Bridging has been proposed for both multivalent metal cations [14], [29] and polyamines [30] as a possible mechanism of aggregation enhancement, whereas cations bridge negatively charged parts of two monomers. The binding region of spermidine has been established as 109–140 aa with NMR by studying complexes of polyamines with alpha-synuclein [31]. The main negatively charged cluster is 124–135 [31] and therefore if we were to detect such bridging we would detect even shorter rupture distances than we observed here. Since the interaction sites are mainly outside the C-terminus it is unlikely that these interactions originate from polyamine bridging between two molecules.

Alternatively, the origin of 36 and 26 nm rupture distances may be incomplete release of long-range intramolecular interactions upon binding of spermidine. Although it is expected to release the C-terminal region from interactions with the rest of the molecule, thus exposing the NAC region, spermidine may not completely eliminate contacts within the C-terminus itself. The existence of residual contacts between stretches 120–130 and 110–115 [28] resulting in relative compactness of C-terminus may very well account for contour length shortening. Importantly, compaction of the C-terminal region has been proposed under conditions that lead to the shielding of negative charges [32]. The monomeric A30P mutant has been shown to have enhanced resistance to pulling compared to WT [33] indicating its higher tendency to form compact structures. This finding may explain our observation of an increased frequency of shorter length rupture events for A30P mutant.

Also, we cannot exclude that the observed short interactions appear due to the expansion of interacting modalities beyond the NAC region. Several studies provided evidence of a preference for β structure outside the NAC region [34], [35]. For example, the 110–120 aa stretch was shown to have a propensity for β structure. The preference for β structure formation coupled with neutralization of nearby negative charges by spermidine preventing intermolecular repulsion might effectively induce interactions between these regions outside the NAC region. Additionally, fibrils of another pathologic mutant, A53T, exhibit an extended β-sheet core with respect to WT α-Syn as detected by ssNMR in the C-terminal region [36]. We hypothesize that such a possibility also exists for the A30P mutant, thus explaining the short distances observed in our study.

The fact that we have observed multiple rupture peaks in addition to single ones further supports our model that the three major interacting sites are of a different nature and that the interacting modalities of alpha-synuclein extend beyond the NAC region. Some of the multiple rupture peaks contain all three major interactions identified for single peaks suggesting a sequential release of the interacting regions. A relatively low abundance of multiple peaks observed in our measurements suggests that dimerization with many sites formed simultaneously is rather unfavorable, perhaps due to steric difficulties that prevent the formation of such a complex dimeric contact. Our data also show that the A30P mutant has a lower propensity for multiple peaks, perhaps due to greater preference of this mutant towards adopting structures that extend into the C-terminal region compared to WT.

The presence of the pathologic single point mutation A30P results in early-onset familial PD cases [24] and was shown to accelerate aggregation of α-Syn *in vitro* [37], [38]. It is still unclear how a single point mutation can affect the aggregation properties of α-Syn. Previous force spectroscopy studies suggested that this mutation drastically shifts the conformational equilibrium to ß-sheet-like conformers [33]. Our data show a strong effect of the A30P mutation on α-Syn misfolding and interactions within the dimer. This effect is quite surprising as the mutation is located rather far from the NAC region (Fig. 6B). This finding suggests that the interactions within the α-Syn misfolded dimer are more complex, and the distant N-terminal region contributes to the protein's misfolding.

### Effect of polyamines on alpha-synuclein conformation

The low propensity of α-Syn monomers to interact at physiologically relevant conditions is in line with our early observations [18], [21] and explains the numerous observations on the slow aggregation kinetics of α-Syn. A conformational flip to a misfolded, aggregation-prone conformation is required for triggering the entire aggregation process [22]. We found that spermidine induces such a conformational change, producing a misfolded conformation of α-Syn with a higher propensity for self-assembly. Various environmental conditions and the presence of external binding partners of α-Syn stimulate the protein's aggregation. Our data presented here suggest that the cellular polyamine spermidine can be another factor triggering a conformational change in α-Syn. Importantly, it has been recently demonstrated that increased levels of higher order polyamines (spermine/spermidine) are directly linked to cellular toxicity of α-Syn and, therefore, PD development [16]. The precise mechanism of how polyamines promote the aggregation of α-Syn is not well understood. Current models explaining the conformational behavior of α-Syn in the presence of polyamines are rather conflicting. Some studies propose that the presence of polyamines stimulates conformational conversion of extended forms into compact states [39]. The higher proportion of such compact conformations induces faster aggregation. Previously, the formation of partially folded intermediate in the presence of metal cations or low pH has been proposed as a major trigger of α-Syn aggregation [40], [41]. Only slight conformational changes were reported to be induced by spermine in a study using FRET [42]. On the other hand, NMR studies and MD simulations suggested that polyamines stimulate the formation of more extended conformations by complexing with the negatively charged C-terminus [31] and releasing long-range contacts between the C-terminal region and NAC and the N-terminal parts of the α-Syn molecule [28].

The controversy may originate from the ensemble nature of the methods used in these studies. Overall conformational preferences induced by the binding of external factors or by environmental conditions may not reflect the actual reason for a higher aggregation rate. The transient formation of misfolded forms of α-Syn may still be a rare event although with a slightly higher probability to form. The formation of misfolded conformers cannot be monitored by ensemble-based methods due to the relatively low contribution of these species to the overall population of conformers. We have shown that AFM force spectroscopy as described in this work is capable of not only detecting misfolded states of α-Syn but also providing a detailed characterization of the protein misfolded states. Importantly, we found that the pathogenic mutation A30P does not increase the propensity of α-Syn to misfold in the presence of spermidine, but it rather increases the strength of interprotein interactions stabilizing the dimer structure. We propose that the mutation affects conformational preferences of monomeric α-Syn distinct from WT as supported by several previous studies [43], [44], [45]. These preferred conformations of the A30P mutant have higher forces of intermolecular interactions according to our data, which might lead to a quicker association of α-Syn molecules.

In summary, we report novel findings that the very first steps of α-Syn self-assembly are severely affected by the presence of the cellular polyamine spermidine. Cellular polyamines, such as spermidine, have multiple essential functions in living organisms, and their levels are maintained via highly regulated pathways. Disturbed metabolism may result in enhanced levels of polyamines. Our data suggest that the presence of spermidine at concentrations comparable to physiological ones induces conformational changes in α-Syn producing a misfolded state of the α-Syn monomer. Therefore, in addition to their normal cellular functions, biogenic polyamines may also have a pathogenic role initializing aggregation of α-Syn. Misfolded states of α-Syn formed in the presence of spermidine are more prone to undergo self-assembly than the normal state of the molecule as detected by single-molecule force spectroscopy. Importantly, we have found that more than one segment within the protein molecule might be responsible for the initial association of α-Syn into dimers and possibly into higher-order oligomers and fibrils. This finding suggests that even the first step of α-Syn self-assembly (dimerization) possesses some degree of heterogeneity. We hypothesize that these different misfolded conformations can lead to different types of oligomers and define the aggregation pathway. The marked differences in the misfolding patterns between WT α-Syn and A30P mutant might be responsible for the higher propensity of the mutant to aggregate and cause early-onset PD.

## Materials and Methods

### Preparation of recombinant α-Syn proteins

A cDNA encoding human α-Syn A30P or A140C was amplified by PCR and subcloned as an Nde I – Hind III fragment into the vector pT7-7, yielding the constructs pT7-A30P and pT7-A140C, respectively. The double mutant A30P-A140C was generated by replacing a 354-base pair fragment from pT7-7 A140C (excised by digesting with Nde I and BamH I) with the equivalent DNA fragment from pT7-A30P. The sequence of the α-Syn-encoding insert in each construct was verified using an Applied Biosystems (ABI 3730 XL) DNA sequencer. Each α-Syn variant was expressed in the E. coli strain BL21 (DE3) and purified using methods adapted from previous studies [46], [47]. Cells were harvested and resuspended in 10 mM Tris-HCl, pH 8.0, 1 mM EDTA, 1 mM DTT, and 1 mM PMSF and stored at−80°C. After one round of freezing and thawing, the cells were lysed with a French pressure cell (Sim Aminco; 1200 psi). Streptomycin sulfate was added to the supernatant (final concentration, 1% (w/v)), and the mixture was incubated on a nutator for 15 min at 4°C. To enrich for α-Syn, the protein mixture was subjected to two successive ammonium sulfate precipitations (the first at 30% saturation, the second at 50% saturation) at 0°C. The pellet recovered after the second precipitation was resuspended in 10 mM Tris, pH 7.4, 1 mM PMSF, and 1 mM DTT. The protein solution was boiled for 5 min, and insoluble proteins were removed by centrifugation at 15,300×g for 10 min. The supernatant was filtered through a 0.22 µm filter and loaded onto a diethylaminoethyl (DEAE) Sepharose FF column (GE healthcare) equilibrated with mobile phase (10 mM Tris, pH 7.4, 5 mM 2-mercaptoethanol). α-Syn was eluted from the column by increasing the concentration of NaCl in the mobile phase from 0 to 1 M over 100 min with a linear gradient. α-Syn eluted at approximately 0.3–0.5 M NaCl. Protein fractions were pooled, and the solution was dialyzed against 10 mM NH_4_HCO_3_ and lyophilized.

### Biochemical characterization of α-Syn variants

To ensure that the A140C and A30P-A140C variants were purified as full-length, intact proteins, each lot of protein was characterized using two methods. As one approach, each protein was analyzed by reacting with 5,5'-dithiobis-(2-nitrobenzoic acid) (DTNB) to determine levels of reduced cysteine [48]. The lyophilized protein was reconstituted in phosphate buffered saline (PBS, 10 mM phosphate buffer, 2.7 mM KCl, and 137 mM NaCl at pH 7.4), centrifuged through a spin-filter with a molecular weight cutoff (MWCO) of 50 kDa or 100 kDa to remove aggregates, and dialyzed against PBS using a Slide-A-Lyzer minidialysis unit (Pierce, MWCO=3,500 Da) at 4°C. The protein was mixed with DTNB in a molar ratio of 1∶1 (36 µM of each) and incubated at 25°C for 5–10 min. From measurements of the absorbance of protein-bound 2-nitro-5- thiobenzoate (TNB) at 412 nm, it was determined that the ratio of cysteine residues to protein molecules was approximately 1∶1. As a second approach, each α-Syn variant was examined by reverse phase high performance liquid chromatography (RP-HPLC) using an Agilent HP1090 system. A RP-HPLC column (Vydac, C18, 4.6 mm i.d. X 250 mm, catalog number 218 TP54) was equilibrated at 1 mL/min with mobile phase (5% (v/v) acetonitrile in 0.1% (v/v) aqueous trifluoroacetic acid at 25°C. The protein was loaded on the column, and bound protein was eluted by increasing the concentration of acetonitrile in the mobile phase from 5% to 100% (v/v) over 60 min. Peak fractions were collected over 42–44 min, pooled, and analyzed by matrix assisted laser desorption ionization (MALDI) mass spectrometry using an Applied Biosystems Voyager System 6270. The mass-to-charge (m/z) values obtained from the MALDI analysis corresponded to the predicted values for full-length α-Syn.

### Tip and mica surface preparation

#### Tip modification

Silicon nitride (Si_3_N_4_) AFM tips (SNL-10, Bruker AFM Probes) were immersed in ethanol for 30 min, rinsed thoroughly with water followed by UV treatment for 30 min (CL-1000 Ultraviolet Crosslinker, UVP, Upland, CA). After the UV treatment, AFM tips were immersed in aqueous solution of 167 μM MAS (maleimide-polyethylene glycol-silatrane was synthesized as described in [20]) for 3 h followed by multiple rinses in water. For covalent attachment of the α-Syn to MAS functionalized tips, a 19 nM protein solution in PBS buffer, was treated with 1 mM DTT (to reduce any disulfide bonds formed between α-Syn molecules) for 10 min and filtered through 10 kDa MWCO centrifuge filter devices (Amicon) 3 times. The protein solution was diluted with PBS buffer to final concentration of 19 nM of α-Syn. MAS functionalized tips were immersed in 19 nM solution of α-Syn for 1 h. After rinsing with PBS buffer, unreacted maleimide moieties were quenched by a treatment with 10 mM β-mercaptoethanol in PBS buffer for 10 min. These functionalized probes were washed with PBS buffer solution and immediately used for force measurements.

#### Surface modification

Mica sheets (Asheville-Schoonmaker Mica Co., Newport News, VA) were cut into ∼1.5 cm×1.5 cm squares and glued onto glass slides using epoxy glue EPO-TEK 353ND (Epoxy Technology, Inc., Billerica, MA). Freshly cleaved mica surfaces were treated with maleimide silatrane (MAS) for 3 hours. MAS modified mica squares were rinsed with water several times to remove non-bound MAS which was followed by a reaction with 19 nM of α-Syn solution in PBS (prepared as described in previous section) for 1 h and rinsed with PBS buffer. Unreacted maleimide moieties were quenched with 10 mM β-mercaptoethanol in PBS buffer for 10 min. The functionalized mica surfaces were thoroughly washed with PBS buffer solution and immediately used for force measurements.

### Force measurements

Force–distance measurements were performed in PBS buffer, at room temperature with the Force Robot 300 (JPK Instruments, Berlin, Germany). Silicon nitride cantilevers with nominal values of spring constants in the range of ∼0.1 N/m were used. Spring constants for each cantilever were obtained using a thermal method with the Force Robot 300 instrument. The ramp size was 200 nm with approach and retraction velocities kept constant at 600 nm/s. A low trigger force (200 pN) was applied. Extension and retraction curves were acquired systematically at different points on the surface set by a grid (5 by 5 µm) where points were separated from each other by 50 nm.

### Data analysis

Thousands of force-distance curves were collected for each alpha-synuclein variant. The curves were filtered using Data Processing software of JPK Force Robot 300 instrument. The curves containing rupture events were fitted with a Worm Like Chain model incorporated into DP-JPK. The persistence length was kept constant at 0.4 nm while the contour length was evaluated as a variable parameter for the best fit of the curve. The persistence length 0.4 nm was chosen because it was found to describe well stretching of a variety of proteins [49]. Measured values of contour length were assembled into statistical histograms and fitted with Gaussian functions to determine maxima of L_C_ distributions.
